# Parents' perceptions of children's emotional well‐being during spring 2020 COVID‐19 restrictions: A qualitative study with parents of young children in England

**DOI:** 10.1111/cch.13034

**Published:** 2022-08-02

**Authors:** Stephanie Chambers, Joanne Clarke, Ruth Kipping, Rebecca Langford, Rachel Brophy, Kim Hannam, Hilary Taylor, Kate Willis, Sharon A. Simpson

**Affiliations:** ^1^ University of Glasgow Glasgow UK; ^2^ University of Birmingham Birmingham UK; ^3^ University of Bristol Bristol UK

**Keywords:** children, COVID‐19, early years, emotional well‐being, nurture, qualitative

## Abstract

**Background:**

During COVID‐19 restrictions in England in spring 2020, early years settings for young children were closed to all but a small percentage of families, social contact was limited and play areas in parks were closed. Concerns were raised about the impact of these restrictions on young children's emotional well‐being. The aim of this study was to explore parents' perceptions of young children's emotional well‐being during these COVID‐19 restrictions.

**Methods:**

We interviewed 20 parents of children 3–4 years due to begin school in England in September 2020. Interviews were conducted via telephone (*n* = 18) and video call (*n* = 2), audio‐recorded and transcribed verbatim. Interviews focused on childcare arrangements, children's behaviour and transition to school. A sample of transcripts were coded line by line to create a coding framework, which was subsequently applied to the remaining transcripts. Coded data were then analysed using a nurture lens to develop themes and further understanding.

**Results:**

Participants were predominantly mothers (*n* = 16), White British (*n* = 10) and educated to degree level (*n* = 13), with half the sample living in the highest deprivation quintile in England (*n* = 10). Five were single parents. Three themes developed from nurturing principles were identified: creating age‐appropriate explanations, understanding children's behaviour and concerns about school transition. Parents reported that their children's emotional well‐being was impacted and described attempts to support their young children while looking ahead to their transition to primary school.

**Conclusions:**

This study is one of the first to examine in‐depth perceptions of COVID‐19 restrictions on young children's emotional well‐being. The longer term impacts are not yet understood. Although young children may be unable to understand in detail what the virus is, they undoubtedly experience the disruption it brings to their lives. The well‐being of families and children needs to be nurtured as they recover from the effects of the pandemic to allow them to thrive.

**Key messages**

- COVID‐19 restrictions have had a negative impact on young children.
- We interviewed parents of children in England due to begin primary school to understand perceptions of children's experiences of COVID‐19 restrictions.
- Nurture principles helped us understand the challenges families faced.
- Key themes identified were creating age‐appropriate explanations, understanding children's behaviour and concerns about school transition.
- We suggest a nurturing approach to recovery to support children and their families.

## 1 INTRODUCTION

The COVID‐19 pandemic has resulted in substantial disruption to daily life for many, including families with young children. In England between March and May 2020, early years childcare settings, hereafter called nurseries, were closed to all except children of essential workers and vulnerable children. There were limits on leaving one's home and accessing play areas in parks, while non‐essential shops were closed and social contact between households forbidden. Nine million jobs were furloughed with the UK Government paying 80% of the salary of employees unable to work (HM Revenue and Customs, 2020).

Nurseries provide a safe place for children to develop and thrive (Melhuish et al., 2015). During COVID‐19 restrictions in Spring 2020, of all children identified as vulnerable in England, only 1 in 20 were attending childcare/schooling, despite being eligible to attend (Department for Education, 2020). In England, more children returned to nurseries in June 2020, although often for fewer days or hours than before (Lawler, 2020). Concerns were raised about the impact of the pandemic on children's health and well‐being, particularly young children's social and emotional well‐being (The Children's Society, 2020). Preschool child development includes learning to play with others (Smith, 1978), cooperation and developing attachments to secondary caregivers (Verschueren & Koomen, 2012). These crucial development opportunities were unavailable for most children during nursery closures. Increased parental stress while educational and community settings were closed has been identified (Benner & Mistry, 2020; Griffith, 2022; Masten & Motti‐Stefanidi, 2020; Spinelli et al., 2020; Yoshikawa et al., 2020). Previous studies have shown that parents experiencing greater stress levels are less likely to engage in responsive care with their children (Guajardo et al., 2009; Östberg, 1998). Responsive care is important for children's development and is likely to be even more essential during disruptions (Yoshikawa et al., 2020). Lee et al. (2022) found that parental social isolation and recent employment loss were positively associated with child maltreatment risk during the early stages of the pandemic among a US sample of parents. A systematic review has also highlighted that the mental health of caregivers was associated with children's mental health outcomes (Naff et al., 2022).

Researchers hypothesized the likely impact of COVID‐19 restrictions on young children based on theory and research from previous emergency situations (Benner & Mistry, 2020; Brown et al., 2020; Masten & Motti‐Stefanidi, 2020; Prime et al., 2020; Yoshikawa et al., 2020). Findings reporting the impact on children's outcomes are only now emerging. Many studies focus on primary and secondary school‐aged children (Larsen et al., 2021; Maftei et al., 2022; Melegari et al., 2021; Royal College of Paediatrics and Child Health Children & Young People's Engagement Team, 2021), and there are only a small number of studies that focus specifically on preschool age children and their families (Stassart et al., 2021). For example, Deoni (2022) found that US children born just before or during the pandemic scored lower in terms of verbal, motor and overall cognitive performance compared with the scores from children born before this time. Italian mothers of preschool children reported poorer sleep quality and greater emotional difficulties in children (Di Giorgio et al., 2021). Egan et al. (2021) interviewed parents of children aged 1–10 years in Ireland (Egan et al., 2021). They reported that children's social lives were disrupted and that children struggled without the usual routines of formal education and childcare. Parents in this study also described increased emotional issues such as tantrums.

The majority of studies emerging are quantitative. For example, an analysis from a cross‐sectional survey with Belgian parents found that parents recognized a range of emotional difficulties in their 4–13 year old children during the pandemic and reported it had negatively impacted family well‐being (Stassart et al., 2021). Public Health Scotland surveyed parents of children aged 2–7 years with half reporting worse behaviour and mood in their children during COVID‐19 restrictions (Public Health Scotland, 2020). Paul et al. (2021) found a greater persistence of reported emotional and behavioural difficulties in children after age two during the pandemic compared with prepandemic reports. Similar results were found among UK parents of preschool children, who reported their concern about the impact of the pandemic on their children's well‐being and wished for greater support in managing children's behaviours and emotions (Dodd et al., 2020).

These studies provide an important overview but do not provide in‐depth understanding of the issues faced by families and the techniques families used to support their preschool children through the pandemic disruption. This study used qualitative interviews to explore parents' perceptions of their preschool children's emotional well‐being during COVID‐19 restrictions. The impact of COVID‐19 on children's social and emotional development will likely be felt by families in the long term. It is essential that we understand families' experiences of this time to support them appropriately and to inform future responses to similar emergency situations.

## 2 METHOD

### 2.1 Research design

The study aim emerged from a wider qualitative study exploring the impact of COVID‐19 restrictions on the health, well‐being and behaviours of children 3–4 years due to begin primary school in September 2020. Impact on eating and activity is reported elsewhere (Clarke et al., 2021). As emotional well‐being is so important at this age (Mihaela, 2015), we sought to analyse these data separately using a nurturing framework to help make sense of the data and identify recommendations.

### 2.2 Recruitment

We recruited parents of preschool children from nurseries in the West Midlands and South West of England participating in a trial led by this study's authors (https://napsaccuk.blogs.bristol.ac.uk/). Nurseries emailed parents to inform them about the study. We also publicized the study via social media sites in these areas. Parents registered their interest via a website and provided demographic information and postcode data to derive deprivation level (https://imd-by-postcode.opendatacommunities.org/imd/2019). We sampled participants from a pool of 85 who had expressed their interest in participating. To promote diversity, we oversampled those living in areas of high deprivation and those who were from non‐White backgrounds. We interviewed all fathers who expressed an interest in participating. We emailed these parents an information sheet and consent form and telephoned to confirm participation and arrange a suitable interview time.

### 2.3 Data collection and analysis

KW, JC and SC conducted 20 interviews via video call (*n* = 2) or by telephone (*n* = 18) after gaining verbal consent. Interviews were audio‐recorded and transcribed verbatim. An overview of the topic guide can be found in Table 1. Participants received a £30 shopping voucher following interview.

**TABLE 1:** Topic guide

Domain	Questions
Emotional well‐being	How has your child been feeling about the coronavirus restrictions?
	What effects (if any) do you think the restrictions have had on your child's well‐being?
	Have the restrictions had any effect on your child's mood and feelings? (positive or negative)
	Have you noticed any changes at all in your child's behaviour? (positive or negative)
	What is the impact on the child of any reduced social contact with other children, friends and family?
Transition to school	How are you feeling about your child starting school in September?
	How is your child feeling?
	Any thoughts about whether child will start in September, or whether you may delay starting school?
	Are there any things you would have been doing to help prepare the child for starting school that you are not able to do because of the disruption? For example, visits to new school and end of preschool special events
	What could help with the transition during this time of uncertainty?

Transcripts were checked for accuracy, anonymized and imported into NVIVO‐12 to facilitate data management and analysis. The study team developed an initial coding frame. Three researchers, JC, RB and HT, coded the same three transcripts line by line and compared their codes, with inconsistencies discussed and appropriate amendments made to the coding framework. The remaining transcripts were single coded by these three researchers with ongoing discussion about amending the framework when new codes emerged. These line‐by‐line codes were then aggregated into broader codes and later used to develop explanatory themes.

### 2.4 Analytical framework

We use nurturing as a lens to examine parenting during COVID‐19 restrictions. Nurture is a recurring theme in the literature on how families might manage their children's well‐being during the pandemic (Brown et al., 2020; Masten & Motti‐Stefanidi, 2020; Prime et al., 2020; Walsh, 2020; Yoshikawa et al., 2020). Nurture has its basis in attachment theory (Bowlby, 1958), which considers the relationship between a child and their primary caregiver. Where children have attached securely to their primary caregiver, there is warmth and responsiveness (Ainsworth et al., 2015). From the strength of this relationship, the child can develop and explore their environment with a feeling of security. However, where this relationship has not been nurtured, the child is more likely to experience social and emotional difficulties and is less likely to form appropriate secondary attachments to further their development and enable them to flourish. Evidence from the literature on emergency situations has consistently stressed the importance of close relationships in supporting children through adversity (Masten & Motti‐Stefanidi, 2020).

**FIGURE 1:**
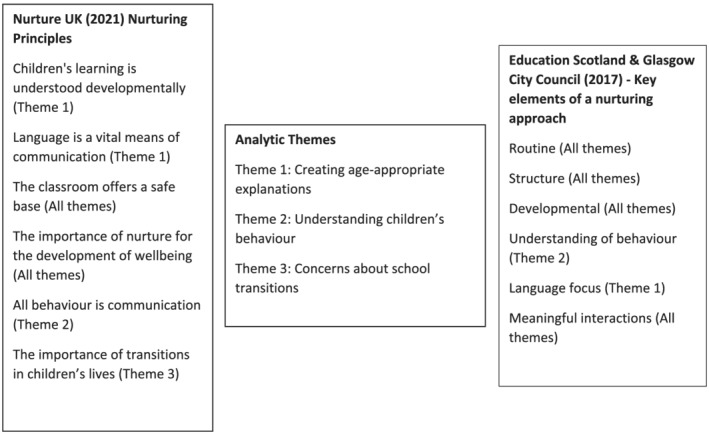
Overview of analytical framework

Key concepts from the literature on nurturing proved useful in understanding parents' accounts and developing our analysis (Dalton et al., 2020). Nurture is a major focus in UK education (Scottish Government, 2021), and given that children were missing out on educational opportunities during pandemic restrictions, we engaged with educational nurturing literature as we analysed our data. We drew on key nurturing principles identified by Nurture UK (2021) and Education Scotland and Glasgow City Council (2017)—frameworks widely adopted by educational organizations and establishments across the United Kingdom. The links between these frameworks and the analytical themes are outlined in Figure 1. Initial codes were regrouped around these nurture concepts by SC and developed into three key themes through which we present our data.

## 3 FINDINGS

Participants were predominantly mothers (*n* = 16), White British (*n* = 10) and educated to degree level (*n* = 13), with half the sample living in the highest deprivation quintile (*n* = 10) (see Table 2). Five were single parents. More than half the sample (*n* = 13) reported that at least one parent was not working during the strictest restrictions, with three reporting no household members in employment at the time of interview.

**TABLE 2:** Participant characteristics

Characteristic categories		*N* (%)
Gender	Female	16 (80)
	Male	4 (20)
Age group	21–25	1 (5)
	26–30	7 (35)
	31–35	5 (25)
	36–40	2 (10)
	41–45	5 (25)
Ethnicity	White	12 (60)
	Asian or Asian British	4 (20)
	Black or black British	1 (5)
	Mixed	3 (15)
IMD quintile (1 = most deprived)	1	10 (50)
	2	2 (10)
	3	3 (15)
	4	3 (15)
	5	2 (10)
Paid employment	Yes	17 (85)
	No	3 (15)
Educated to degree level	Yes	13 (65)
	No	7 (35)
Single parent	Yes	5 (25)
	No	15 (75)

Routine and structure are key components within nurture frameworks (Education Scotland & Glasgow City Council, 2017; Nurture UK, 2021), and during non‐pandemic times, their implementation supports educational establishments to become safe spaces for children. The pandemic disrupted this sense of safety, and parents highlighted the challenges that this uncertainty created within their families. Children's prepandemic routines were described by parents as being important for the development of their children's health and well‐being. We present parents' perceptions of their children's Covid‐19 experiences through three main themes. These incorporate nurture principles/key elements of a nurturing approach and relate to the overarching issues of structure, routine, safety, development, interaction and health and well‐being. The three themes are (1) creating age‐appropriate explanations, (2) understanding children's behaviour and (3) concerns about school transitions.

### 3.1 Creating age‐appropriate explanations

The sudden onset of COVID‐19 restrictions left parents to explain an entirely new and potentially alarming situation to their preschool child. There were three main areas parents had to manage in terms of supporting their child's understanding of COVID‐19 and restrictions. The first related to what the virus was, transmission and infection, and the hygiene practices necessary to avoid infection. As routines changed and children heard news reports, parents described their anxieties in relation to their children's understanding of what was going on.

He hears things, just through the TV, and especially during the beginning of the pandemic, he was hearing every day how many people had caught the virus and how many people had died from the virus … I ended up turning the TV off, so he could not hear it … a child should not worry about things like that. (Mother_12)

Parents attempted to explain what was happening in a way that a preschool child could understand. Parents reported that their children had assimilated the new vocabulary (e.g. ‘lockdown’, ‘coronavirus’ and ‘germs’) and information about hygiene measures when parents presented this information at an appropriate developmental level. They also accepted however that their child's understanding of the situation was superficial because of their age.

With having a four‐ and a three‐year‐old, they are not going to comprehend what COVID‐19 is. So we talked about there are germs and we have got to stay inside to stay safe, you have to wash your hands when mummy tells you to. It was very difficult for them to process … It's too much for a toddler to comprehend. (Mother_13)

The second issue parents faced was explaining why certain favoured places, like nurseries and play areas in parks, were no longer accessible. These places were tied to children's routines, and therefore, parents described their children not only missing these locations but also the day‐to‐day experiences that provided fun and certainty in their lives.

I think that's what he found difficult, is that everything looks the same and everyone seems the same, but he could not do the same things that he normally does. (Mother_6)

The third issue was ceasing in‐person contact with those who were important to their children, such as grandparents and friends. Parents described this as one of the most difficult restrictions for children to understand. What had previously been close, nurturing relationships had to be reconceptualized as potentially dangerous and to be avoided.

I think it made him really sad, not being able to give his grandma a cuddle. He just could not quite comprehend why he could not do that, even though he knew about the virus and he knew that it can be dangerous, and we are quite open with the boys and we explained to them how severe it can be for some people. (Mother_6)

### 3.2 Understanding children's behaviour

Parents reported negative changes in their child's mood or behaviour during the period of restrictions, including increased tantrums and anger; unsettled, anxious and emotional behaviours; and developmental regression (e.g. sleep and independence). Parents reported children displaying shock, anger, frustration and sadness at the new restrictions placed upon their lives. Parents generally sought to understand why their children were acting in this way, recognizing the emotional impact of change in routine, lack of stimulation, not going out as much, reduced social contact and anxiety about coronavirus.

He was really emotional. He used to play up a lot. We had no routine, so he had no structure. He was so energetic, I could not burn all his energy off. (Mother_10)

Although lack of routine was identified by parents as an explanation for changes in children's behaviour, they found it challenging to create new routines. There were limited opportunities to do this without the support parents previously relied upon, such as childcare, local amenities, family and friends. To reduce the impact of children missing grandparents and others, parents attempted to connect them via technology. While this was described as positive for a small number of children, for others this was not a means through which they could connect adequately.

When we have tried to video call some of [my daughter's] friends or family, she would just get upset, it wasn't a good enough substitute. (Mother_14)

Nearly half of parents reported changes in their child's sleep patterns during restrictions. The main issue reported was difficulty in getting children to fall sleep. Parents often perceived this as an indirect impact of restrictions on their children. Disrupted sleep was viewed as a consequence of reduced physical exertion and increased screen time and emotional issues such as boredom and anxiety.

We noticed some changes in his sleep pattern, really. Yes, I can say that even in his sleep, sometimes he's restless. He's got nightmares. He leaves his bed to come and sleep with us. (Mother_1)

One parent commented that reduced sleep impacted on her child's behaviour, indicating the circularity of the challenges families were facing at this time.

The longer that [COVID‐restrictions have] gone on, like she does not go to sleep at night, and then she's already irritable before she's even started the next day. (Mother_5)

Some parents reported positive changes in their child's behaviour and mood during the restrictions, which they identified as due to spending more time with the immediate family. These parents reported their children felt secure at home with them and that this appeared to make them happier.

I think she's been a little bit closer to us because having to spend a little bit more time with us … I think in that aspect it's been good that we have seen her grow and she appreciated the time with us. (Father_2)

One parent found a positive impact from having more time to set a sleep routine.

I work all the time so we hardly got to see each other … I was just throwing him into bed at any time … Obviously, having lockdown, I had time to do stuff before bedtime. We started having a bath at the same time every night, then story time and then sleep time, which I've stuck to. (Mother_10)

### 3.3 Concerns about school transition

Parents recognized starting primary school is a major transition for any child; however, they viewed it as a normative transition. It was an expected change in routine that may or may not raise general anxieties for families. Some parents talked about their child seeming ‘ready’ for school. For some, there was also a sense of relief that school—and a daily routine—would soon begin.

He will start in September, so that will start as a new routine for him … I think we all need it. I think we all need time away from each other. I think it's not healthy being around each other all the time. (Mother_7)

Nevertheless, many parents admitted to some concerns about this transition. Some of this was a natural response to their child transitioning to the next stage in life, for example, around toilet training. However, some felt the context of the COVID‐19 pandemic made this transition, and their feelings toward it, more complicated. Many normal transition activities (such as in school visits, stay‐and‐play sessions and contacts with teachers) had been delayed, done ‘virtually’ or cancelled altogether. Some parents reported that schools had been proactive in trying to accommodate transition activities in a way that was COVID‐secure, such as through virtual contacts.

I think the school have done a really good job. They have sent some videos out that are on YouTube that we have watched which have been really helpful. (Father_2)

Other parents had not had this experience and were concerned about how they would adequately prepare children for school.

I just think the transition is going to be harder because of lockdown, because we have not had as much communication with the school as we possibly would have had … I think that's definitely affected the transition for both parents and the child, because if the parents have got no information, they cannot impart anything to the child. (Mother_3)

Unease due to uncertainty was discussed by many parents. The myriad of unknowns articulated included how to safely drop children at school, where children would eat, the extent to which social distancing would be in place and whether education would be full time. Many parents therefore expressed anxiety about what autumn/winter would bring.

How is life going to change for our school child and the circumstances? If another second wave comes then how will life be like in December? … Will we be going back to lockdown in December or what will happen during the school time as well? (Mother 11)

## 4 DISCUSSION

Parents in this study perceived that COVID‐19 restrictions had impacted their preschool children's emotional well‐being and they described attempts to nurture and attend responsively to their children's needs. The findings outlined the ways in which parents tried to communicate what was happening to their children in a way that was developmentally appropriate and by using language that their child would understand. Most parents reported negative changes in their child's behaviour and throughout the interviews sought to understand what their children were trying to communicate through this behaviour. They described the behaviours as understandable responses to lack of routine, boredom and anxiety. Parents' options for alleviating these emotions were limited and almost totally reliant on skilfully navigating life at home without external support.

Parents recognized how important starting school was in their children's development. The importance of transition is recognized in nurturing frameworks (Education Scotland & Glasgow City Council, 2017; Nurture UK, 2021) and educational establishments try to support transitions by adequately preparing children through transition activities. Parents reflected on the absence of these activities, and in the context of the pandemic, they were unsure how best to prepare children. The potential impact of a lack of sufficient transition has been recognized (Bowyer‐Crane et al., 2021). Research with teachers has outlined the barriers in supporting nursery to school transition during COVID‐19 restrictions, with skills regression a substantial challenge to be addressed, particularly when working with families from disadvantaged backgrounds and children with special educational needs and disability (Bakopoulou, 2021).

Our findings highlight how parents appeared to incorporate nurturing principles and elements to protect and reassure their children during their experiences of pandemic life. Masten and Motti‐Stefanidi (2020) argue family routines provide safety and comfort to children experiencing severe disruption. A small number of families in our study said they and their children had experienced some positive benefits during the pandemic, such as having an opportunity to bond. A survey of UK parents with young children identified many benefits of spending more time together during the pandemic (Ipsos MORI, 2020). Yoshikawa et al. (2020) hypothesize that increased family closeness may be an important protective outcome of COVID‐19 restrictions.

It was clear, however, that families in this study found it challenging to replace normal routines that were usually scaffolded by preschool, work, children's activities and socializing. Previous studies of family resilience have stressed the importance of relational approaches where parents are supported by extended family, community‐based resources and wider societal structures (Walsh, 2020). During the COVID‐19 restrictions, external sources of nurturing care were largely absent for the parents in this study. Instead, parents had to focus nurturing care through their relationship with their child, and highlighted their anxieties about their children's well‐being despite their attempts. The WHO and UNICEF have developed a framework of *Nurturing Care for Early Childhood Development* (World Health Organization & UNICEF, 2018) which highlights the importance of environments that enable nurturing care including enabling policies, supportive services, empowering communities and caregivers' capabilities.

Walsh (2020) suggests the best way for parents to support children through ongoing COVID‐19 disruption is by creating meaning from challenges, a hopeful outlook and the creation of a sense of purpose. The parents we interviewed were reflecting on a relatively short time frame of only a few months and therefore may not have anticipated that restrictions would continue beyond 2020. In early 2021, restrictions were extended, and it is likely that the resilience described by these families was tested, particularly in the winter months, without the wider support of external community resources (Norris et al., 2008). Remote learning was implemented once again for the majority of school‐aged children across the United Kingdom in January and February 2021; however, in England, early years establishments largely remained open. This policy decision perhaps recognized the immense strain families with young children faced in trying to care for them under previous restrictions.

Family stress is a particular concern for child welfare during emergency situations and appears to have had an impact on wider families' experiences during the pandemic. Quantitative surveys of parents have indicated that higher levels of parental mental illness during the pandemic were negatively associated with responsive parenting practices (Brown et al., 2020; Romero et al., 2020; Westrupp et al., 2021). A major concern has been that the rate of child abuse in England rose during restrictions. In 2020–2021, serious incident notifications were 19% higher compared with 2019–2020. The number of serious incident notifications relating to child deaths increased by 35 compared with the same period in the previous year, and notifications relating to serious harm increased by 31 (UK Government, 2022).

### 4.1 Strengths and limitations

This study offers novel and in‐depth insights into how parents with a preschool child managed their children's emotional well‐being in response to COVID‐19 restrictions. It complements quantitative surveys (Dodd et al., 2020; Public Health Scotland, 2020) by providing in‐depth understanding of the stressors parents and their children face. These findings will be important for improving the support provided to families during future restrictions and informing plans for longer term recovery (Hefferon et al., 2020). We note however that we only include the perspectives of parents, rather than the children themselves.

Using a nurturing lens helped to develop the themes identified at an earlier analysis stage and provided interpretative insight into early child development and responsive care. This allowed us to move beyond a solely descriptive analysis to find that parents were engaging in positive parenting practices that could be described as responsive and nurturing. The analytic frameworks used helped to operationalize some of the theoretical concepts included in the literature early in the pandemic that hypothesized some of the likely impacts of restrictions on families (Masten & Motti‐Stefanidi, 2020; Prime et al., 2020; Walsh, 2020; Yoshikawa et al., 2020). We acknowledge, however, that parents who were struggling to care for their children during COVID‐19 restrictions might be less likely to participate in a study such as this, or to disclose less optimal parenting strategies in the interview context. The focus on emotional well‐being, both in the study and through the nurturing lens in the analysis, meant that we are unable to present evidence on families' pandemic experiences in relation to material resources. In the interviews, we did not ask parents about financial hardship, and none of them raised this spontaneously during the discussions. Changes in income levels due to the pandemic may have impacted families' well‐being substantially, and this is an area that requires further study.

The study had strong representation of families living in areas of high deprivation and of different ethnicities, providing the opportunity to ensure multiple perspectives were considered that can often be marginalized in academic research. Nevertheless, the sample was highly educated and may not reflect the experiences of parents with lower levels of education. Another study limitation was that it only focused on one aspect of family life at this time, specifically parents' experiences of their preschool child's emotional well‐being. There was only limited time within the interview to discuss wider family life, including parents' emotional well‐being and wider family relationships, which are also likely to impact on young children's emotional well‐being.

### 4.2 Implications and conclusion

This study is one of the first to provide an in‐depth understanding of parental perceptions of young children's emotional well‐being under COVID‐19 restrictions. With that in mind, it has relevance to families and the professionals who support families globally. Although young children may be unable to understand in substantial detail what the virus is, they undoubtedly experience the monumental disruption it brings to their young lives. Routines that brought them comfort and security were disrupted overnight, with only their parents available to support them.

The long‐term impacts of this disruption so early in life are not yet understood, but studies examining families' experiences at this time will provide a foundation to build appropriate support for the future. The nurturing focus of this study has highlighted the importance of creating secure environments for children to thrive in. Nurture is a possible mitigation strategy to reduce the negative impact of the pandemic on children's well‐being. It can also be extended to form the basis of recovery too, including a coordinated response from services and decision‐makers to support families to create strong, positive relationships and understand behaviour as communicating important information about a child's emotional well‐being. Suggested responses thus far have included extending the school day or reducing the length of school holidays to allow children to catch up on lost learning. These recovery options do not acknowledge the need for children's well‐being to be addressed first, as is embedded in nurture approaches in educational settings, before learning can take place optimally. We therefore propose that communities, services and decision‐makers work to nurture children and their families' well‐being. This will help parents to offer a complementary responsive approach to their children as they support them through and beyond the COVID‐19 pandemic.

## CONFLICT OF INTEREST

The authors declare they have no conflicts of interest.

## ETHICS STATEMENT

Ethical approval for the study was granted by University of Bristol Faculty of Health Sciences Research Ethics Committee (Ref: 106002). Participant consent was recorded on a separate audio file before the interview commenced.

## AUTHOR CONTRIBUTIONS

The study was conceived by JC, RK, SC, KW, HT, RB, KH, SAS and RL. JC led the study with oversight from RL and RK. Interviews were conducted by JC, SC and KW. Coding of the data was performed by JC, HT and RB. SC produced the first draft of the manuscript, with all other authors providing critical review and intellectual content. All authors read and approved the final manuscript.

## Data Availability

Anonymized study data will be made available under controlled access via the University of Bristol data repository. Requests for controlled data will be referred to an appropriate Data Access Committee for approval before data can be shared with bona fide researchers, after their host institution has signed a Data Access Agreement.
